# The Effect of Compensatory Strategies in Reducing to Postoperative Airway Invasion, and Pharyngeal Residue in Patients Undergoing Esophagectomy for Esophageal Cancer

**DOI:** 10.1007/s00455-025-10885-5

**Published:** 2025-10-14

**Authors:** Asako Kaneoka, Haruhi Inokuchi, Rumi Ueha, Taku Sato, Takao Goto, Koichi Yagi, Yasuyuki Seto, Nobuhiko Haga

**Affiliations:** 1 The University of Tokyo Hospital Rehabilitation Center, 7-3-1 Hongo Bunkyo-Ku, Tokyo, 113-8655 Japan; 2 The University of Tokyo Hospital Swallowing Center, 7-3-1 Hongo Bunkyo-Ku, Tokyo, 113-8655 Japan; 3 Department of Otolaryngology and Head and Neck Surgery, The University of Tokyo, 7-3-1 Hongo Bunkyo-Ku, Tokyo, 113-8655 Japan; 4 Department of Gastrointestinal Surgery, The University of Tokyo, 7-3-1 Hongo Bunkyo-Ku, Tokyo, 113-8655 Japan; 5 National Cancer Center Hospital, 5-1-1 Tsukiji, Chuo-ku, Tokyo, 104-0045 Japan; 6 National Rehabilitation Center for Persons with Disabilities, 4-1 Namiki, Tokorozawa, 359-8555 Saitama Japan

**Keywords:** Esophageal cancer, Esophagectomy, Dysphagia, Liquid modification, Chin-tuck posture

## Abstract

Few studies have reported the effects of dysphagia management after esophagectomy in esophageal cancer. We aimed to investigate the impact of liquid modification and chin-tuck posture (head flexion) on reducing airway invasion and pharyngeal residue after esophagectomy. We included patients who underwent esophagectomy between June 2017 and January 2020. A videofluoroscopic swallowing study (VFSS) was conducted on 5 ml liquid boluses with four viscosities: thin, mildly thickened, moderately thickened, and extremely thickened, all administered in a neutral head position. Trials with thin and moderately thickened liquid were repeated with head flexion. The penetration-aspiration scale (PAS) scores and residue grades were compared across viscosities and head positions. Thirty-three patients met the inclusion criteria (mean age 65.3 ± 9.0 years). Moderately and extremely thickened liquids resulted in less frequent penetration or aspiration compared to thin liquids, while mildly thickened liquids showed no significant difference in airway protection. Residue grades did not differ significantly across the viscosities tested. Head flexion did not affect the occurrence of aspiration or residue grades compared with the neutral head position for thin and moderately thickened liquids. Penetration on thin liquid was less frequent in head flexion than in the neutral position. Liquid modification improved swallowing safety by reducing penetration and aspiration without affecting pharyngeal clearance. While the chin-tuck posture did not significantly reduce aspiration, its potential to lessen penetration suggests a role in airway protection that warrants further study. These findings warrant validation in larger studies.

## Background

Esophageal cancer is the eighth most prevalent cancer and the sixth leading cause of cancer-related mortality worldwide [1]. Radical resection of esophageal cancer, or esophagectomy, remains the primary treatment approach [2] but is associated with significant complications in approximately 25% of patients [3]. Critical complications include anastomotic leakage, pulmonary complications, strictures, reflux, damage to the recurrent laryngeal nerves, dysphagia, and gastrointestinal symptoms [4]. Among these, dysphagia is particularly significant because of its association with an increased risk of pneumonia and mortality after esophagectomy [5]. Thus, a comprehensive assessment and interventions are crucial for enhancing patient outcomes and quality of life.

Post-esophagectomy swallowing deficits arise from multiple pathological changes, including vocal fold immobility due to recurrent laryngeal nerve damage [6–9], delayed onset of swallowing from laryngeal sensory impairment [8], reduced tongue base retraction [6] and pharyngeal constriction [6, 10] from preoperative muscle weakness, and reduced hyolaryngeal elevation caused by postoperative scarring and adhesion, particularly after thoracic and cervical lymph node dissection [6–8, 11, 12]. These impairments could contribute to penetration/aspiration and post-swallowing pharyngeal residue, increasing pneumonia risk [6, 9]. Despite the clinical significance, only a few studies have examined the effects of post-esophagectomy swallowing management [5], leaving the effectiveness of interventions on post-esophagectomy airway invasion and pharyngeal residue uncertain.

One strategy to improve airway protection is the chin-tuck posture, which modifies bolus flow by repositioning the head or neck [13]. Specifically, head flexion (C1–C2) and neck flexion (C3–C7) achieved by tucking the chin have distinct effects on swallowing physiology [14]. In healthy adults, head flexion decreases the distance between the tongue base and posterior pharyngeal wall, reduces the vallecular space, and narrows the laryngeal inlet [15], enhancing airway protection. Conversely, neck flexion prolongs the tongue base contact with the posterior pharyngeal wall [15]and increases the duration and distance of the upper esophageal sphincter (UES) opening compared to the neutral head position [16], promoting more efficient bolus passage.

The effects of neck flexion have been observed in post-esophagectomy patients. Kumai et al. [10] investigated the impact of the chin-down posture (defined as neck flexion) on pharyngeal residue in 14 patients who had undergone esophagectomy with three-field lymphadenectomy, findings that neck flexion significantly increased the UES opening diameter and duration, prolonged laryngeal vestibule closure, and reduced pyriform sinus residue. However, the study did not focus on the effect of head flexion on airway protection.

Another common strategy to mitigate airway invasion is liquid modification, which slows bolus flow, as thin liquids flow rapidly and require intact sensory input and timely coordination of the swallowing muscles [17]. In contrast, thickened liquids slow bolus flow, allowing more time for airway closure and reducing the likelihood of aspiration [18]. Despite its widespread use, the effect of liquid modification has been validated in only one randomized controlled trial that also examined the impact of the “chin-down” posture. Logemann et al. compared nectar-thickened (300 cps = 300 mPa·s), honey-thickened (3,000 cps = 3,000 mPa·s), and a chin-down posture in patients with oropharyngeal dysphagia due to dementia or Parkinson’s disease [13]. Honey-thickened liquids most effectively reduced aspiration, but the study underscored the need for further investigation into the effects of liquid modification on pharyngeal residue and its applicability to dysphagic populations with different underlying etiologies. Notably, the impact of liquid modification has not been specifically examined in patients with post-esophagectomy dysphagia [19]. Given the unique physiological changes following esophagectomy, it remains unclear whether the benefits of liquid modification extend to this patient population.

We aimed to examine the effects of liquid modification and the chin-tuck posture, specifically head flexion, on reducing airway invasion and pharyngeal residue in patients who have undergone esophagectomy. We hypothesized that liquid modification and head flexion would prevent penetration/aspiration while potentially increasing pharyngeal residue.

## Methods

### Setting

This retrospective observational study was conducted at a teaching hospital in Tokyo, Japan. Written informed consent was obtained from all participants, and the Institutional Review Board approved the study protocol (Ref. No. 2018016NI).

### Participants

Patients who underwent curative esophagectomy for esophageal cancer between June 2017 and January 2020 were recruited. The inclusion criteria were patients who underwent both laryngoscopic examination to assess vocal fold function and videofluoroscopic swallowing study (VFSS) for the first time since surgery, at two weeks postoperation. The exclusion criteria included dysphagia identified in the preoperative VFSS, an inability to provide informed consent, and an incomplete VFSS protocol due to examiner discretion following the observation of penetration-aspiration events to ensure patient safety.

### VFSS Protocol

The VFSS was conducted by otolaryngologists in lateral projection with patients in an upright position at 1/30-second intervals. Considering the pulmonary toxicity of aspirated barium sulfate [20], a hypoosmotic, nonionic, iodinated contrast agent (Iohexol; Omnipaque, Daiichi Sankyo Co., Ltd., Tokyo, Japan) was used and administered using a syringe. The protocol has been employed in our institution for many years, particularly for elderly and critically ill patients, and was approved by the institutional ethics review board. Patients completed one trial of 5 ml of contrast agent with four different viscosity levels using a xanthan gum-based thickening agent (Toromeiku^®^ SP; Meiji Co., Ltd., Tokyo, Japan) [21]: thin, mildly thickened (50–150 mPa·s), moderately thickened (150–300 mPa·s), and extremely thickened (300–500 mPa·s) as per the national standard for dysphagia diet [22]. The contrast agent was prepared based on prior rheological assessments conducted at our institution to ensure compliance with this standard. Initially, patients were in the neutral head position, being guided to “swallow with the neck in a comfortable position, as you usually do.” They then swallowed the following four liquid consistencies: 1 st : moderately thickened, 2nd : extremely thickened, 3rd : mildly thickened, and 4th : thin. This order was maintained consistently for all participants. Trials with thin and moderately thickened liquid were repeated with head flexion (C1–C2), guiding patients to “tuck your chin, and then swallow in that position.” Head flexion (C1–C2) was further adjusted as needed and confirmed visually on the VFSS monitor by consensus among the three otolaryngologists present during the examination. The examiners ensured that the patients maintained a head flexion while swallowing. To minimize the impact of any pharyngeal residue from the previous trial, if residue was present, patients were instructed to perform additional swallows, or if necessary, suction was performed to clear the residue before proceeding to the subsequent trial.

### Data Collection

Demographic data, disease status, and treatment characteristics were extracted from the electronic medical records. The laryngoscopic examination records were used to determine the presence or absence of vocal fold immobility. The VFSS movie files were deidentified and archived in a hospital data storage system for subsequent blinded analyses. Two assessors, a board-certified speech-language therapist and a physiatrist, independently rated the participants’ levels of airway invasion using the penetration-aspiration scale [23] (PAS score: 1, normal; 8, material enters the airway and passes below the vocal folds and no effort is made to eject it), noting the timing of penetration/aspiration before, during, and after swallowing [24]. Pharyngeal residue severity was assessed using the Functional Dysphagia Scale (score: 0, no residue; 3, >50% of the width of the structure) [25] or the vallecula and pyriform sinuses after the first swallow attempt. Disagreements between the assessors were resolved through discussion, and the residue score was determined by consensus between the two raters.

### Statistical Analyses

Descriptive statistics were calculated for the continuous variables (means and standard deviations), ordinal (medians and ranges), and categorical (counts and percentages) variables. The PAS scores were categorized into three levels of airway protection: normal (PAS 1, 2), penetration (PAS 3, 4, 5), and aspiration (PAS 6, 7, 8). The effects of liquid modification on the levels of airway protection were analyzed using Fisher’s exact test. Similarly, the effect of liquid modification on pharyngeal residue scores in the vallecula and pyriform sinuses was analyzed using the Friedman’s test, and when significant, post-hoc pairwise comparisons were conducted using Wilcoxon signed-rank tests with Bonferroni correction.

The impact of head position (neutral vs. flexion) on the levels of airway protection for thin and moderately thickened liquids was analyzed using Fisher’s exact test. Likewise, the impact of head position on pharyngeal residue scores in the vallecula and pyriform sinuses was analyzed using Friedman’s test. Statistical analyses were performed using SAS (version 9.4; SAS Institute, Inc., Cary, NC) and GraphPad Prism (version 10.0; GraphPad Software, San Diego, CA). Statistical significance was set at *p* < 0.05.

## Results

### Participant Characteristics

During the study period, 61 patients underwent subtotal esophagectomy. Twenty-eight patients were excluded because both laryngoscopic examination and VFSS data were not available as required by the study protocol. Consequently, 33 patients were included in the analyses.

Table 1 shows the participant characteristics. The mean age of the participants was 65.3 ± 9.0 years, and 27 (81.8%) were male. Most participants underwent curative esophagectomy for thoracic cancer, followed by reconstruction with a gastric tube conduit and cervical anastomosis, typically via the posterior mediastinal route. Regarding the surgical approach, seven patients (21.2%) underwent transthoracic esophagectomy, while 26 (78.8%) underwent mediastinoscopic esophagectomy, a minimally invasive procedure performed via a transcervical and transhiatal approach. Among these, 22 procedures (84.6%) were performed with transhiatal robotic assistance.

**Table 1:** Participant characteristics (*n* = 33)

Characteristics	Mean ± SD	*n* (%)
Age (years)	65.3 ± 9.0	
*Gender*		
Male		27 (81.8)
Female		6 (18.2)
*Location of cancer*		
Cervical		0
Thoracic		31 (93.9)
Abdominal		2 (6.1)
*Histology*		
Squamous cell carcinoma		33 (100.0)
Adenocarcinoma		0 (0.0)
*Clinical stage* ^*^		
Ⅰ		5 (15.2)
Ⅱ		19 (57.6)
Ⅲ		8 (24.2)
Ⅳ		1 (3.0)
*Neoadjuvant chemotherapy*		
Yes		20 (60.6)
No		13 (39.4)
*Surgical procedure*		
Mediastinoscopy		26 (79.8)
Thoracotomy		7 (21.2)
*Reconstruction route*		
Retrosternal		1 (3.0)
Posterior mediastinal		32 (97.0)
*Reconstruction organ*		
Gastric		32 (97.0)
Colon		1 (3.0)
*Anastomosis*		
Cervical		32 (97.0)
Intrathoracic		1 (3.0)
*Lymph node dissection*		
Two-field		3 (9.0)
Three-field		30 (91.0)
*Jejunostomy*		
Yes		7 (21.2)
No		26 (78.8)
*Postoperative complications*		
Anastomotic leakage		3 (9.0)
Vocal fold immobility		26 (78.8)
Aspiration pneumonia**		6 (18.2)

^*^The TNM Classification of Malignant Tumors (UICC 8th edition)

^**^Among the six patients who developed postoperative pneumonia, three exhibited aspiration (PAS 7–8) in the head-neutral position during VFSS

Despite variations in surgical procedures, all participants had their sternohyoid and sternothyroid muscles divided, and lymph node dissection was performed. Partial or total immobility of vocal folds was observed in 26 participants (78.8%) during the postoperative laryngoscopic examination. Of those, 23 (88.5%) exhibited left unilateral vocal fold immobility, while three (11.5%) had bilateral vocal fold immobility. The average period from surgery to postoperative VFSS was 15.5 ± 6.0 days.

### Effect of Liquid Modification

On thin liquid swallows, 25 patients (75.7%) were normal, while five (15.2%) presented penetration, and three (9.1%) presented aspiration. All penetration/aspiration events occurred “during” swallowing.

The impact of liquid modification on airway protection is shown in Table 2. A significant difference in airway protection was observed across the liquid viscosity levels (*p* = 0.0073). Post-hoc pairwise analyses indicated no significant difference between thin and mildly thickened liquid (*p* = 0.76). However, significant differences were found between thin and moderately thickened liquids (*p* = 0.03) and between thin and extremely thickened liquids (*p* = 0.01).

**Table 2:** Penetration and aspiration events by liquid viscosity levels in head neutral position

Liquid viscosity		Thin	Mildly thickened (50–150 mPa·s,)	Moderately thickened (150–300 mPa·s)	Extremely Thickened (300–500 mPa·s)	*p*-value
*Airway protection*						
Normal		25 (75.7)	27 (81.8)	32 (97.0)	33 (100.0)	
Penetration		5 (15.2)	5 (15.2)	1 (3.0)	0 (0.0)	
Aspiration		3 (9.1)	1 (3.0)	0 (0.0)	0 (0.0)	
Total		33 (100.0)	33 (100.0)	33 (100.0)	33 (100.0)	0.0073

Data are presented as count (%). The Fisher’s Exact Test

The effect of liquid modification on pharyngeal residue was presented for the vallecula (Table 3) and pyriform sinuses (Table 4). There was no significant difference in the residue grade of the vallecula (*p* = 0.50) or pyriform sinuses (*p* = 0.16) across the different liquid viscosity levels.

**Table 3:** Residue scores for vallecula by liquid viscosity levels in head neutral position

Liquid viscosity		Thin	Mildly thickened	Moderately thickened	Extremely thickened	*p*-value
*Residue score*						
0		4 (12.1)	8 (24.2)	9 (27.3)	9 (27.3)	
1		17 (51.5)	11 (33.3)	12 (36.4)	8 (24.2)	
2		9 (27.3)	11 (33.3)	8 (24.2)	12 (36.4)	
3		3 (9.1)	3 (9.1)	4 (12.1)	4 (12.1)	
Total		33 (100.0)	33 (100.0)	33 (100.0)	33 (100.0)	0.50

Data are presented as count (%). The Friedman’s test

Residue scale: 0 = no residue; 1 = < 10% of the width of valleculae or pyriform sinuses; 2 = 10–50% of the width; 3 = > 50% of the width

**Table 4:** Residue scores for pyriform sinus by liquid viscosity levels in head neutral position

Liquid viscosity		Thin	Mildly thickened	Moderately thickened	Extremely thickened	*p*-value
*Residue score*						
0		19 (57.6)	21 (63.6)	22 (66.6)	23 (69.7)	
1		10 (30.3)	9 (27.3)	7 (21.2)	9 (27.3)	
2		4 (12.1)	3 (9.1)	2 (6.1)	1 (3.0)	
3		0 (0.0)	0	2 (6.1)	0 (0.0)	
Total		33 (100.0)	33 (100.0)	33 (100.0)	33 (100.0)	0.16

Data are presented as count (%). The Friedman’s test

### Effect of Head Flexion

The impact of head flexion on airway protection is shown in Table 5. In the head neutral position, there were three aspiration events in thin liquids (9.1%), while there were none in moderately thickened liquids (0.0%). There was no significant difference in the levels of airway protection for thin liquid swallows (*p* = 0.28) or moderately thickened liquid swallows (*p* = 0.28) between neutral head position and head flexion. However, penetration events on thin liquid were less frequent in head flexion (3.0%) than in the neutral position (15.2%).

**Table 5:** Penetration and aspiration events by head position

Liquid viscosity Head position		Thin		*p*-value	Moderately thickened		*p*-value
		Neutral	Flexion		Neutral	Flexion	
*Airway protection*							
Normal		25 (75.7)	28 (85.0)		32 (97.0)	32 (97.0)	
Penetration		5 (15.2)	1 (3.0)		1 (3.0)	1 (3.0)	
Aspiration		3 (9.1)	4 (12.0)		0	0	
Total		33 (100.0)	33 (100.0)	0.28	33 (100.0)	33 (100.0)	0.28

Data are presented as count (%). The Fisher’s Exact Test

The effects of head flexion on residue grade in the vallecula and pyriform sinuses are presented in Tables 6 and 7, respectively. There was no statistically significant difference in residue grade for the vallecula (*p* = 0.07) or pyriform sinuses (*p* = 1.00) between the neutral and head flexion positions for thin liquid swallows. Similarly, no significant difference was found in residue grade for the vallecula (*p* = 0.23) or pyriform sinuses (*p* = 0.24) between the two head positions for moderately thickened liquid swallows. However, there was a trend toward a lower incidence of Grade 3 residue, the most severe grade, in the head flexion position compared to the neutral position. Specifically, in the thin liquid trials, the number of participants with Grade 3 residue in the vallecula decreased from 3 (9.1%) in the neutral position to 0 (0.0%) in the head flexion position. Similarly, in the moderately thickened liquid trials, the number of participants with Grade 3 residue in the vallecula decreased from 4 (12.4%) in the neutral position to 0 in the head flexion position. In the thin liquid trials, the number of participants with Grade 3 residue in the pyriform sinus increased slightly from 0 to 1 in the head flexion position. However, in the moderately thickened liquid trials, the number of participants with Grade 3 residue in the pyriform sinus decreased from 2 (6.1%) in the neutral position to 0 (0.0%) in the head flexion position.

**Table 6:** Residue rating in the vallecula by head position

Liquid viscosity Head position		Thin		*p*-value	Moderately thickened		*p*-value
		Neutral	Flexion		Neutral	Flexion	
*Residue score*							
0		4 (12.1)	6 (18.2)		9 (27.3)	8 (24.2)	
1		17 (51.5)	17 (51.5)		12 (36.4)	16 (48.5)	
2		9 (27.3)	10 (30.3)		8 (24.2)	9 (27.3)	
3		3 (9.1)	0 (0.0)		4 (12.1)	0 (0.0)	
Total		33 (100.0)	33 (100.0)	0.07	33 (100.0)	33 (100.0)	0.23

Data are presented as count (%). The Friedman’s test

**Table 7:** Residue rating in the pyriform sinus by head position

Liquid viscosity Head position		Thin		*p*-value	Moderately thickened		*p*- value
		Neutral	Flexion		Neutral	Flexion	
Residue score							
0		19 (57.6)	19 (57.6)		22 (66.6)	23 (69.7)	
1		10 (30.3)	10 (30.3)		7 (21.2)	9 (27.3)	
2		4 (12.1)	3 (9.1)		2 (6.1)	1 (3.0)	
3		0 (0.0)	1 (3.0)		2 (6.1)	0	
Total		33 (100.0)	33 (100.0)	1.00	33 (100.0)	33 (100.0)	0.24

Data are presented as count (%). The Friedman’s test

## Discussion

Liquid modification enhanced swallowing safety after esophagectomy by reducing penetration/aspiration without significantly affecting pharyngeal clearance. In contrast, the chin-tuck posture, which is defined as head flexion, did not show statistically significant benefits in alleviating airway invasion or pharyngeal residue. These results suggest that liquid modification may be more effective than head flexion as a compensatory strategy for patients with dysphagia after esophagectomy.

Our findings demonstrate the superiority of thickened liquids over head flexion in enhancing airway protection. This is consistent with Logemann’s investigation, which compared these interventions in patients with dysphagia due to dementia or Parkinson’s disease [13]. Several post-esophagectomy physiological changes in swallowing have been identified, potentially resulting from surgical effects, such as adhesion formation, scarring at the anastomosis, and inflammation [11]. Specifically, delayed swallowing reflex [8], reduced laryngeal elevation [8, 12, 26], and vocal fold immobility [9, 26, 27] are associated with post-esophagectomy aspiration. Furthermore, penetration-aspiration events predominantly occur during laryngeal elevation [6, 12]. In our cohort, more than 75% of patients demonstrated vocal fold immobility, which may partly explain the effectiveness of thickened liquids in reducing airway invasion. Moreover, all penetration-aspiration events occurred during swallowing, supporting the hypothesis that impaired glottic and laryngeal closure—rather than impaired UES opening with overflow of post-swallow residue—was the predominant mechanism underlying aspiration in this population. Taken together with previous findings, these observations suggest that delayed and incomplete airway closure is the primary cause of airway invasion after esophagectomy, and that increasing bolus viscosity, which provides more time to achieve glottic closure, may effectively compensate for these pathological characteristics.

Our study also demonstrated that thickened liquids did not increase pharyngeal residue in the vallecular and pyriform sinus despite previous concerns about using thickened liquids in patients with reduced bolus propulsion [13]. Potential causes of vallecular residue after esophagectomy include reduced tongue pressure [28]and tongue base retraction [6], delayed or incomplete epiglottic inversion [8], and weak pharyngeal contractions [6]. Similarly, the leading causes of pyriform sinus residue in patients undergoing esophagectomy are weak pharyngeal contraction [10], reduced UES opening [10], and reduced laryngeal elevation [6, 11, 12]. Therefore, we initially hypothesized that swallowing thicker liquids would be more difficult for patients undergoing esophagectomy because of the greater pressure required to clear the bolus [17]. Although our hypothesis was rejected, the finding that thickened liquids do not negatively affect pharyngeal clearance is valuable for dysphagia management in esophagectomy patients, given the significant association between pharyngeal residue and postoperative aspiration pneumonia [6]. However, providing thickened liquids has been associated with adverse events such as dehydration, pneumonia, hospitalization, reduced quality of life, aspiration, and decreased intake [19]. Thus, it is clinically essential to weigh the risks and benefits of mitigating potential adverse outcomes.

In contrast to liquid modification, our study did not support the benefits of the chin-tuck posture, defined as head flexion, in improving swallowing safety in patients after esophagectomy. Lewin et al. reported that the chin-tuck posture effectively eliminated aspiration in 17 out of 21 (81.0%) aspirators of thin liquids [19, 29]. Several factors may explain this discrepancy between studies. First, the patient characteristics and surgical procedures may differ. Lewin’s study lacked detailed information on the surgical procedures that affect laryngeal elevation after esophagectomy, such as lymphadenectomy [11] and reconstruction routes [7]. Therefore, the postoperative swallowing pathophysiology may differ from that observed in the present study. Additionally, the methods for the chin-tuck posture may vary, as there was no description of how the participants were instructed to tuck their chin or whether the investigators aimed for head or neck flexion. Because head and neck flexion produce different effects [15], this variation may influence the effectiveness of airway invasion management. Although no significant reduction in aspiration was observed in our cohort, penetration events appeared less frequent with head flexion than with the neutral position. This finding suggests a potential benefit of the chin-tuck posture in reducing penetration, which warrants further investigation.

Our study did not align with Kumai et al. [10], who reported that the chin-tuck posture reduced pyriform sinus residue after esophagectomy with three-field lymphadenectomy, while vallecular residue remained unchanged. This discrepancy between studies may have arisen from differences in head positioning (head flexion vs. neck flexion). Since our research aimed to reduce airway invasion during laryngeal elevation, we guided the patients to flex their heads. Thus, depending on the target symptoms, such as airway invasion or pharyngeal residue, selecting either posture (head or neck flexion) may be necessary. Although statistical significance was not reached, our results indicated a trend toward reduced Grade 3 vallecular and pyriform sinus residue with head flexion. This suggests that head flexion may have a potential role in alleviating severe residue, which warrants further investigation in larger cohorts.

This study had several limitations common to retrospective observational studies [30]. First, the findings were based on a small sample size from a single-center setting, which limits generalizability. For example, although there was no statistically significant difference in residue grade between the neutral and head flexion positions, our findings suggest a potential clinical benefit of head flexion in reducing severe post-swallow residue. Further investigation with a larger sample size is warranted to confirm these trends. Second, our inclusion criteria potentially excluded patients with severe medical conditions or prolonged serious dysphagia who could not undergo VFSS, thus limiting variability in the study participants. This was because, for safety reasons, VFSS had to be discontinued in patients who exhibited aspiration or penetration and were unable to eject the aspirated materials. If patients with severe dysphagia had been included, it is likely that not only aspiration during swallowing but also increased vallecular and pyriform sinus residue would have been observed, leading to a higher risk of post-swallow aspiration after swallowing. In such case, the potential clinical benefit of head flexion in reducing severe post-swallow residue suggested in our data might have been more clearly demonstrated. Third, for safety reasons, the analysis was restricted to swallowing only 5 mL of liquid due to missing data from VFSS discontinuation. Larger liquid volumes may reveal more penetration and aspiration events, providing additional insights into dysphagia after esophagectomy. Similarly, the effectiveness of the thickening liquid or chin-tuck posture may differ for larger bolus volumes because the UES opening is volume-dependent [31]. Fourth, the order of bolus presentation could not be randomized, causing a potential order effect. However, to minimize observer bias, three otolaryngologists performed the VFSS, and the two raters were blinded to both patient background information and bolus viscosity when assessing the PAS and residue grade. Fifth, this study did not analyze the pathophysiology of swallowing. The effects of bolus thickening and head position may vary depending on the presence or absence of impairments beyond vocal fold immobility, such as reduced pharyngeal contractility, reduced upper esophageal sphincter (UES) opening, and delayed pharyngeal swallow. Future studies should include a detailed assessment of pharyngeal phase impairments to better understand how these factors influence swallowing outcomes. Finally, this study presents data collected at two weeks post-esophagectomy. We believe that the findings primarily reflect postoperative decompensation rather than the long-term effects of esophagectomy. However, as this study lacked long-term follow-up, assessing long-term effects is necessary for future research. Despite these limitations, this study provides valuable insights into postoperative dysphagia management and future investigations aimed at providing appropriate interventions for patients undergoing esophagectomy.

## Conclusion

This study demonstrated that liquid modification may improve swallowing safety after esophagectomy by reducing penetration and aspiration without significantly affecting pharyngeal clearance. The chin-tuck posture (head flexion) did not significantly reduce aspiration, though fewer penetration events suggest a potential role in airway protection. Thickened liquids appear useful as a compensatory strategy, especially for patients with posterior mediastinal reconstruction and cervical anastomosis without severe aspiration, but larger studies are warranted.

## Data Availability

Data sets generated during the current study are available from the corresponding author upon reasonable request.
